# Optimization of Kombucha Fermentation from Green Tea and Pineapple Juice: Chemical, Physicochemical, and Bioactive Profiles for Functional Beverage Development

**DOI:** 10.1021/acsomega.6c03893

**Published:** 2026-05-14

**Authors:** Socorro Vanesca Frota Gaban, Samylla Sáthilla Queiroz Nogueira, Francisco Marlon Mota Marques, Chiara Porro, Elenilson G. Alves Filho, Lorena Mara A. Silva, Kirley M. Canuto

**Affiliations:** † Department of Food Engineering, Federal University of Ceara, Bloco 858, Campus do Pici, CEP, 60440-900 Fortaleza, Ceará, Brazil; ‡ Department of Clinical and Experimental Medicine, University of Foggia, 71121 Foggia, Italy; § 196588 Embrapa Agroindústria Tropical, Rua Doutora Sara Mesquita, n. 2270, Pici, CEP, 60511-110 Fortaleza, CE, Brazil

## Abstract

This study evaluated the metabolic evolution and functional quality of green tea-based kombucha and pineapple juice-based kombucha fermented with the same symbiotic culture of bacteria and yeasts. Beverages were monitored over 0, 3, 5, and 7 days of fermentation using nuclear magnetic resonance spectroscopy alongside complementary physicochemical and color analyses to elucidate changes in organic acids, amino acids, and phenolic compounds. Both beverages showed progressive pH reduction to approximately 3.4, increased titratable acidity, and lightning of color (increased L, decreased *a* and b*) over time. Sucrose content decreased progressively in both systems, accompanied by ethanol production followed by oxidation to acetic, gluconic, and succinic acids, which increased significantly (*p* < 0.05). Pineapple juice-based kombucha exhibited higher total acid production and faster acidification, while green tea kombucha maintained greater stability in malic and citric acids. Among nitrogenous and phenolic compounds, epicatechin gallate (ECG) and epigallocatechin-3-gallate (EGCG) peaked at day 5 before declining due to oxidative degradation; theanine and alanine showed transient increases, indicating microbial synthesis and assimilation. Caffeine remained relatively stable throughout fermentation. Antioxidant activity measured by the ABTS assay revealed significantly higher radical scavenging capacity in green tea kombucha (10.36 μmol TE/g at day 0 to 10.33 μmol TE/g at day 7) compared to pineapple kombucha (1.90 μmol TE/g to 3.00 μmol TE/g over the same period). Overall, day 5 was identified as the optimal fermentation point, balancing acid production, pH reduction, and preservation of bioactive compounds, supporting the development of functional kombucha beverages from both tea and fruit matrices.

## Graphical abstract

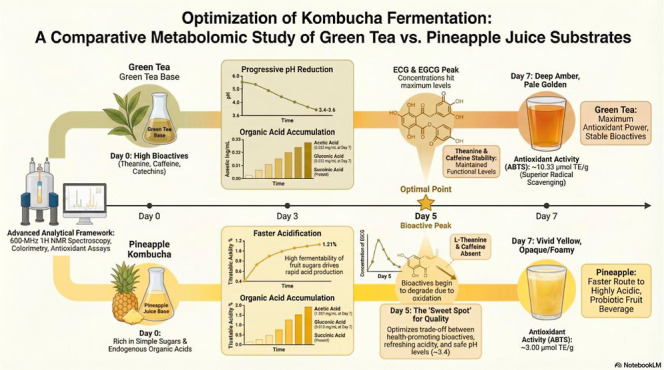

## Introduction

Kombucha is a traditional fermented beverage obtained from the symbiotic metabolism of bacteria and yeasts (SCOBYs) in a sweetened tea infusion.^1^ During fermentation, a complex microbial consortium, composed mainly of *Komagataeibacter*, *Gluconobacter*, and *Saccharomyces* species, converts sugars to organic acids, ethanol, carbon dioxide, and a wide variety of secondary metabolites, including polyphenols, vitamins, and bioactive peptides.^2^ This transformation not only contributes to the characteristic sour and refreshing flavor of the drink but also enhances its functional properties, making kombucha an increasingly attractive product for both the beverage industry and the health-food market.^2,3^

The composition of kombucha is influenced by several factors, including the substrate type, fermentation time, temperature, and microbial activity.^1^ Traditionally prepared with black or green tea (*Camellia sinensis*), the beverage contains catechins and methylxanthines, such as caffeine and theanine, which contribute to its antioxidant potential and physiological benefits. However, recent studies have explored the use of alternative substrates, such as fruit juices and herbal infusions, to expand the sensory diversity and functional profile of kombucha. These new formulations not only appeal to consumers seeking natural and tropical flavors but also provide fermentable sugars and additional phytochemicals that can modulate the metabolic pathways of microorganisms involved.^4,5^

Pineapple (*Ananas comosus*), for instance, is a promising substrate for kombucha fermentation due to its high content of simple sugars, organic acids (citric and malic), phenolic compounds, and bioactive enzymes such as bromelain.^6^ Its natural composition supports rapid microbial growth and acid production, resulting in a beverage with distinctive sensory and nutritional properties. When fermented with the kombucha symbiotic culture of bacteria and yeasts (SCOBY), pineapple juice undergoes both yeast-driven alcoholic fermentation and bacterial oxidation of ethanol and glucose, leading to the production of key metabolites such as acetic, gluconic, and succinic acids. These compounds not only regulate the beverage’s acidity and flavor but also exert beneficial physiological effects, including antimicrobial, antioxidant, and detoxifying actions.^7–9^

Organic acids play a pivotal role in the chemical and sensorial identity of kombucha. Acetic acid is the principal acid responsible for the drink’s sharp taste and preservative properties, while gluconic and glucuronic acids contribute to its detoxifying potential and metabolic regulation.^10,11^ Succinic and malic acids, on the other hand, are intermediates of the tricarboxylic acid (TCA) cycle and reflect the balance between yeast and bacterial metabolism. Monitoring the kinetics of these compounds provides critical insights into the fermentation dynamics, particularly in mixed-substrate systems combining tea and fruit matrices. Moreover, the accumulation of these acids is closely linked to pH reduction, titratable acidity, and microbial succession, which together determine product safety, sensory acceptance, and shelf stability.^10,11^

In parallel, the transformation of bioactive compounds during kombucha fermentation is also significant. Catechins, especially epicatechin gallate (ECG) and epigallocatechin gallate (EGCG), undergo enzymatic and oxidative reactions that can lead to degradation or conversion to simpler phenolics with enhanced bioavailability. Theanine and caffeine, characteristic of green tea, may also be modulated by microbial metabolism, influencing both flavor and neuroactive potential. In fruit-based systems, such as pineapple kombucha, microbial biotransformation may result in the liberation or synthesis of phenolic acids such as gallic and ferulic acids, enhancing the antioxidant and health-promoting profiles of the final beverage.^12,13^

Despite the growing number of studies on kombucha, there remains a lack of an integrated understanding regarding how different substrates influence the balance between organic acid production and the preservation of bioactive compounds during fermentation. Excessive fermentation times often lead to overacidification, loss of polyphenols, and unfavorable sensory changes, while insufficient fermentation may result in poor microbial stability and a reduced functional value. Therefore, identifying an optimal fermentation time is critical for achieving the best trade-off among acidity, pH, and bioactive retention.^14^

In this context, the present study aimed to compare the metabolic evolution of green tea-based kombucha and pineapple-based kombucha fermented with the same SCOBY using nuclear magnetic resonance (NMR) spectroscopy and complementary physicochemical analyses. The quantitative profiles of key organic acids (acetic, gluconic, succinic, malic, citric, gallic, and formic acids) and bioactive compounds (theanine, alanine, epicatechin gallate, epigallocatechin-3-gallate, and caffeine) were evaluated over a 7-day fermentation period. These data were integrated with changes in pH, titratable acidity, and color parameters to elucidate the relationship among microbial metabolism, chemical composition, and functional quality of the beverages.

On the basis of the integration of metabolic and physicochemical findings, this research sought to establish the fermentation stage that maximizes bioactive compound retention while ensuring adequate acid development and product safety. The comparative approach between green tea- and pineapple-based kombucha provides novel insights into the technological potential of mixed fermentations and supports the development of new functional beverages with diversified sensory and nutritional profiles.

## Materials and Methods

### Raw Materials

Pineapple (*A. comosus*), water, green tea, and sugar (Parceria Alimentos Ltd., Ceará, Brazil) were purchased from local markets in Fortaleza, Ceará State, Brazil. The SCOBY was obtained from the Functional Nutrition Laboratory of UFC.

### Inoculum Preparation

For inoculum preparation, *C. sinensis* leaves (0.5% w/v) were maintained at 100 °C for 10 min. The tea was filtered through a nylon sieve, glucose was added to a final concentration of 100 g/L, and SCOBY was mixed in the solution. The mixture was maintained for 10 days at 25 °C.^1^

### Preparation of Conventional Kombucha and Pineapple Kombucha-Like Beverage

To prepare fruit juices, the fruits were washed and sanitized with a sanitizer wash (sodium hypochlorite solution) according to the manufacturer’s instructions. Pineapple juice was prepared by mixing 1 kg of pulp with 1 kg of filtered water. The mixture was then filtered and weighed to add 10% sugar. Subsequently, SCOBY was added at a ratio of approximately 300 g to 1 L of fruit juice or tea. The formulas were stored in glass fermenters covered with cotton cloth for 10 days at 19 °C. During this period, each day for the analysis, the fermented beverages were filtered, transferred to amber bottles, and stored under refrigeration.

### Physicochemical Parameters

The kombucha was subjected to pH, obtained by a pH meter (DLA-pH, DEL LAB), and total soluble solids (TSS) using a refractometer (Grandindex Benchtop Digital, model RSG-100ATC) using the methods adopted by the Association of Official Analytical Chemists.^15^ Titratable acidity was determined by titrating 100 mL samples against 0.1 N NaOH.^16^

### Color Measurements

Color was determined using a Hunter Labscan colorimeter (Hunter Associates Laboratory, Inc., Reston, VA, USA) equipped with EasyMatch QC software version 4.1. The results are expressed in CIELAB coordinates of lightness (L*), greenness–redness (a*), and blueness–yellowness (b*).^17^

### Nuclear Magnetic Resonance Analysis

An aliquot of 165 μL of each kombucha sample was mixed with 400 μL of D_2_O (99.9%) and with 35 μL of D_2_O solution containing 14 mM of EDTA (to minimize the ionic strength effect on frequency shifts) and 1% of sodium-3-trimethylsilyl propionate (TMSP-*d*_4_) as an internal standard (signal at δ 0.0). The resultant samples were transferred to 5 mm NMR tubes, and the NMR experiments were performed on an Agilent 600-MHz spectrometer equipped with a 5 mm (^1^H–^19^F/^15^N–^31^P) inverse detection One Probe.

The ^1^H NMR spectra were acquired under quantitative conditions in triplicate using the PRESAT pulse sequence for water suppression at δ 4.79, at a controlled temperature of 298 K. After 5 min for temperature stabilization between the samples and NMR probe, the probe was properly tuned and matched, and then the hard pulse was calibrated to 90° (8.5 μs pulse length at 58 dB of power). The inversion recovery pulse sequence was used to ensure 99.9% relaxation of the ^1^H nuclei (equivalent to 7 times T_1_), which was distributed between the relaxation delay of 18.0 s and the acquisition time of 5.0 s. A total of 32 scans were applied for the 1H NMR analyses under a fixed receiver gain of 32 for all the spectra acquisition, with 48 k of time domain points and a spectral window of 16.0 ppm. In addition, the affected ^1^H NMR region by the signal saturation from the nondeuterated water was also determined before the compound quantification.^18–20^

For ^1^H NMR spectra processing, free induction decays were multiplied by an exponential function equivalent to 0.3 Hz line-broadening before applying the Fourier transform for 32 k points. Phase correction was manually performed, and the automatic baseline correction using polynomial degree 5 was applied over the entire spectral range. The identification of the constituents in green tea and kombucha samples was performed through 2D-NMR evaluation using correlation spectroscopy (COSY), heteronuclear single quantum coherence, heteronuclear multiple bond correlation, assessments using an open-access database (www.hmdb.ca), and literature reports.^21–24^

### Quantification by ^1^H NMR

The compounds with nonoverlapping signals in the ^1^H NMR spectra were quantified by the external reference method provided by the VnmJ 4.2 software used for the NMR spectra acquisitions.^25^ The concentrations were evaluated by the analysis of variance (ANOVA) single factor using the Origin 9.4 software, and the Tukey test was applied to statistically certify the differences between the samples at a significance level of 0.05. The Levene test was applied to test the variance homogeneity between the types of samples.

### Determination of Antioxidant Activity by ABTS Assay

The ABTS radical scavenging assay was performed according to the method described by Re et al.^26^ The absorbance of the samples was measured at 734 nm using a spectrophotometer. Trolox (100–2000 μM) was used as the standard for calibration. The percentage inhibition of the ABTS radical is calculated using eq 1:

1 ABTSscavengingactivity(%)=[(AB−AA)/AB]×100

where *AB* represents the absorbance of the ABTS radical solution with ethanol (control) and *AA* represents the absorbance of the ABTS radical solution in the presence of the sample or standard extract.

### Statistical Analysis

All experiments were conducted in triplicate, and the data are presented as mean ± standard deviation. Statistical analyses were performed using GraphPad Prism (version 9.5.1, GraphPad Software, San Diego, CA, USA). For univariate comparisons, one-way ANOVA followed by Tukey’s post hoc test was used to evaluate differences among fermentation times.

## Results and Discussion

### Physicochemical Parameters

During fermentation, both beverages exhibited marked changes in pH, acidity, and TSS (Table 1). In kombucha, the initial pH of 7.02 ± 0.04 decreased sharply to 3.45 ± 0.02 by day 7, accompanied by an increase in TA from 0.04% to 0.21%, reflecting the formation of organic acids (Table 1).^2,3^

**Table 1:** Physico-Chemical and Color Analysis of Kombucha and Pineapple Juice-Based Kombucha (*n* = 3)

	days			
	0	3	5	7
Kombucha				
pH	7.02 ± 0.04	3.54 ± 0.02	3.50 ± 0.02	3.45 ± 0.02
acidity (%)	0.04 ± 0.01	0.16 ± 0.01	0.20 ± 0.01	0.21 ± 0.01
total soluble solids (°brix)	9.90 ± 0.06	9.40 ± 0.06	9.20 ± 0.21	9.30 ± 0.06
L*	78.98 ± 0.03	91.3 ± 0.05	91.89 ± 0.10	91.61 ± 0.05
a*	6.18 ± 0.01	–0.03 ± 0.01	–0.18 ± 0.04	–0.33 ± 0.02
b*	51.24 ± 0.01	23.99 ± 0.02	23.91 ± 0.15	22.13 ± 0.03
density	1.039 ± 0.00	1.038 ± 0.00	1.037 ± 0.00	1.036 ± 0.00
pineapple juice-based kombucha				
pH	3.83 ± 0.01	3.29 ± 0.01	3.33 ± 0.01	3.68 ± 0.02
acidity (%)	0.41 ± 0.01	0.75 ± 0.03	1.10 ± 0.01	1.21 ± 0.01
total soluble solids (°Brix)	13.4 ± 0.15	11.7 ± 0.06	11.1 ± 0.17	10.3 ± 0.35
L*	25.31 ± 0.35	25.28 ± 0.05	27.4 ± 0.21	29.04 ± 0.42
a*	–2.28 ± 0.01	–1.93 ± 0.05	–1.79 ± 0.03	–2.82 ± 0.06
b*	3.3 ± 0.10	3.74 ± 0.11	3.07 ± 0.14	2.37 ± 0.07
density	1.045 ± 0.00	1.045 ± 0.00	1.042 ± 0.00	1.037 ± 0.00

The TSS showed a slight reduction from 9.90°Brix to 9.30°Brix, suggesting moderate sugar consumption. The density decreased marginally, confirming the gradual conversion of sugars into ethanol and acids.^14^ In pineapple juice-based kombucha, the pH was already acidic at the beginning (3.83 ± 0.01) and remained relatively stable (3.68 ± 0.02) by day 7 (Table 1). However, the acidity increased substantially, from 0.41% to 1.21%, evidencing intense acidification (Table 1). The TSS decreased from 13.4°Brix to 10.3°Brix, and the density decreased correspondingly, confirming active fermentation. These findings indicate that while both fermentations led to acidification, pineapple juice fermentation was characterized by faster sugar metabolism and greater acid production, likely due to the higher availability of simple sugars and the initial presence of organic acids from the fruit.^6^

The marked decrease in pH and increase in acidity observed in both fermentations are consistent with the metabolic activity of acetic acid bacteria and yeasts during kombucha fermentation.^8^ The oxidation of ethanol and glucose into acetic, gluconic, and succinic acids by *Komagataeibacter* and *Gluconobacter* species leads to progressive acidification, which stabilizes the beverage and enhances its preservative and sensory properties.^2,3^ The faster drop in sugar content and higher acidity in pineapple juice-based kombucha can be attributed to the high fermentability of fruit sugars (glucose and fructose) and the presence of endogenous organic acids.^27^

### Color Parameters

The instrumental color analysis (Table 1) and visual observation (Figure 1A,B) demonstrated distinct color modifications throughout fermentation. For kombucha, the lightness (L)* increased from 78.98 to 91.61, while a* values became less positive (from 6.18 to −0.33) and b* values decreased (from 51.24 to 22.13) (Table 1). These changes correspond to visible lightning and loss of reddish-brown tones, confirmed by the photographs: the beverage changed from a deep amber color at day 0 to a paler golden hue after 7 days (Figure 1A). The reduction in a* and b* values could be associated with the degradation of pigments due to oxidation and microbial metabolism during fermentation.^28^ In contrast, pineapple juice-based kombucha showed an increase in L* from 25.31 to 29.04, and a progressive reduction in a* (−2.28 → −2.92) and b* (3.34 → 2.37), producing a beverage that evolved from vivid yellow at day 0 to a paler, slightly opaque yellow with surface foam at day 7 (Table 1).

**Figure 1:**
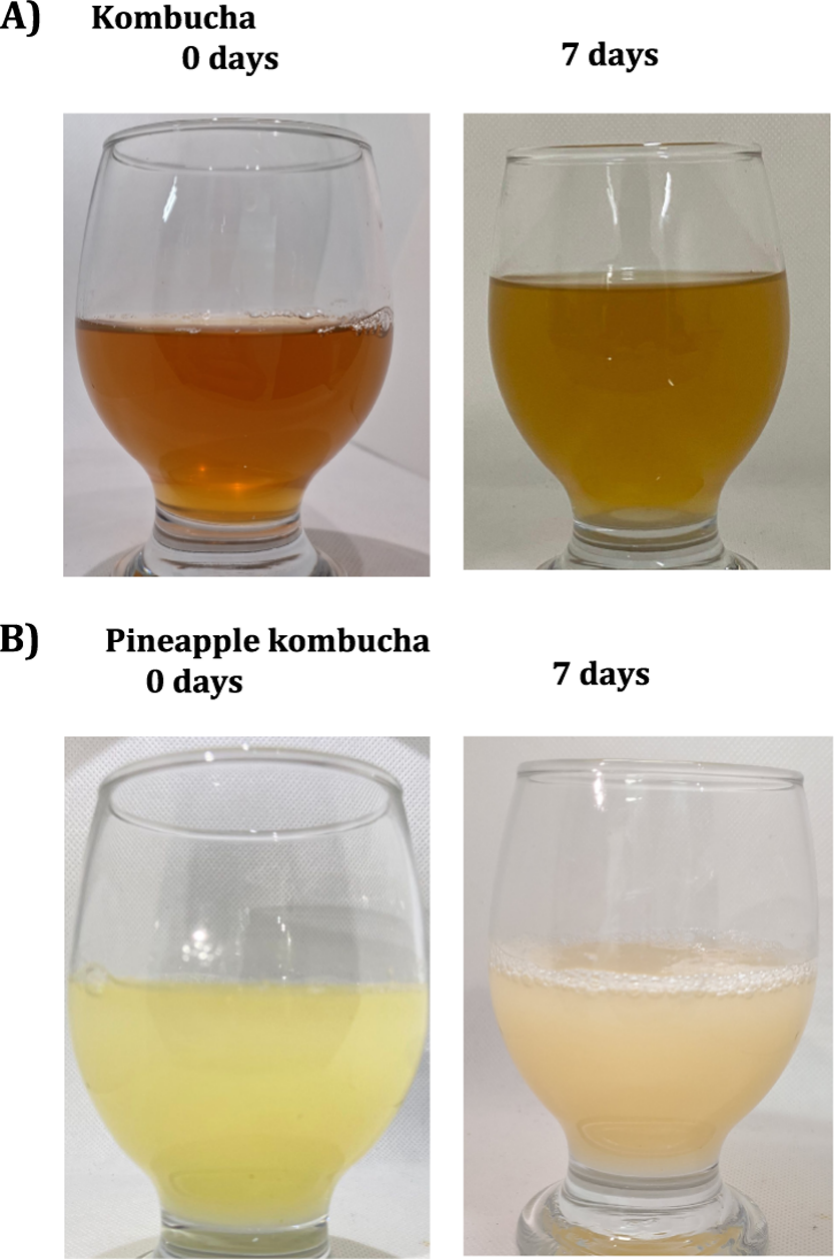
Visual appearance of kombucha (**A**) and pineapple juice-based kombucha (**B**) at 0 and 7 days of fermentation.

Color alterations correspond to the oxidation and polymerization of polyphenolic compounds. In kombucha, catechins and their gallates (EGCG and ECG) undergo enzymatic oxidation to theaflavins and thearubigins, resulting in the lightning of the tea color and reduction in redness and yellowness values.^28^ Similarly, in pineapple juice-based kombucha, browning reactions and oxidation of phenolic acids and carotenoids contribute to the loss of brightness and the development of turbidity.^29^ The slight opacity and foam observed after 7 days arise from yeast proliferation, CO_2_ formation, and biofilm fragments detached from the SCOBY, which is typical of active secondary fermentation.^30^

Overall, both fermentations demonstrated the dynamic transformation of physicochemical and color parameters, confirming that the substrate composition strongly influences microbial metabolism and product characteristics.^5,31^

### Sucrose and Ethanol Dynamics during Fermentation

Figure 2 presents the ^1^H NMR spectra from kombucha at different fermentation stages (0, 3, 5, and 7 days), highlighting compounds such as caffeine, epigallocatechin (EGC), and epigallocatechin-3-gallate (EGCG). In contrast, Figure 3 showcases the ^1^H NMR spectra from pineapple juice-based kombucha during the same fermentation periods. The NMR parameters for the compound’s characterization are made available in the Supporting Information. Figure 4 shows the quantitative variation of sucrose and ethanol during fermentation in both green tea-based kombucha (black bars) and pineapple-based kombucha (gray bars).

**Figure 2:**
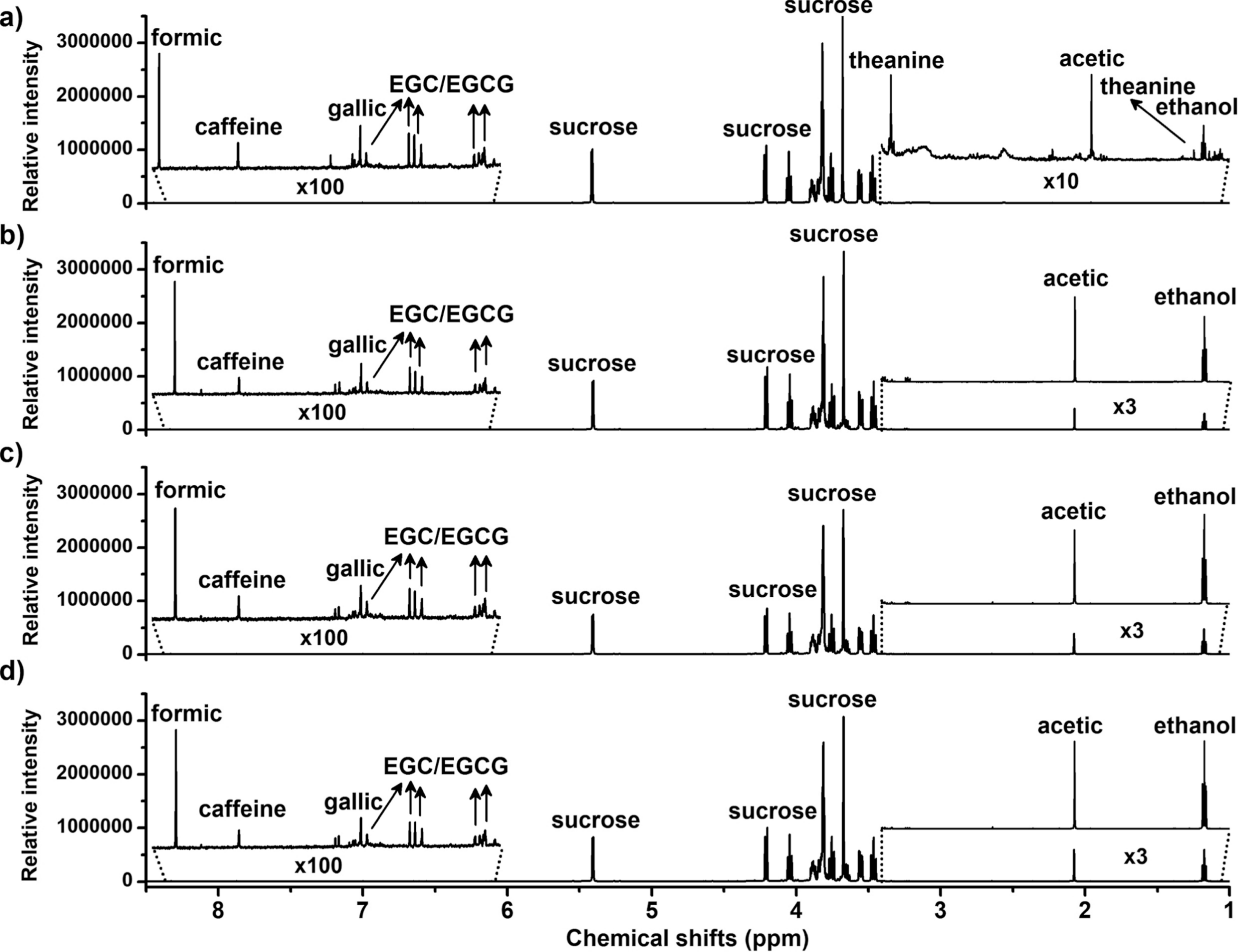
^1^H NMR spectra from kombucha during fermentation (0, 3, 5, and 7 days). EGC, Epigallocatechin; EGCG, epigallo-catechin-3-gallate.

**Figure 3:**
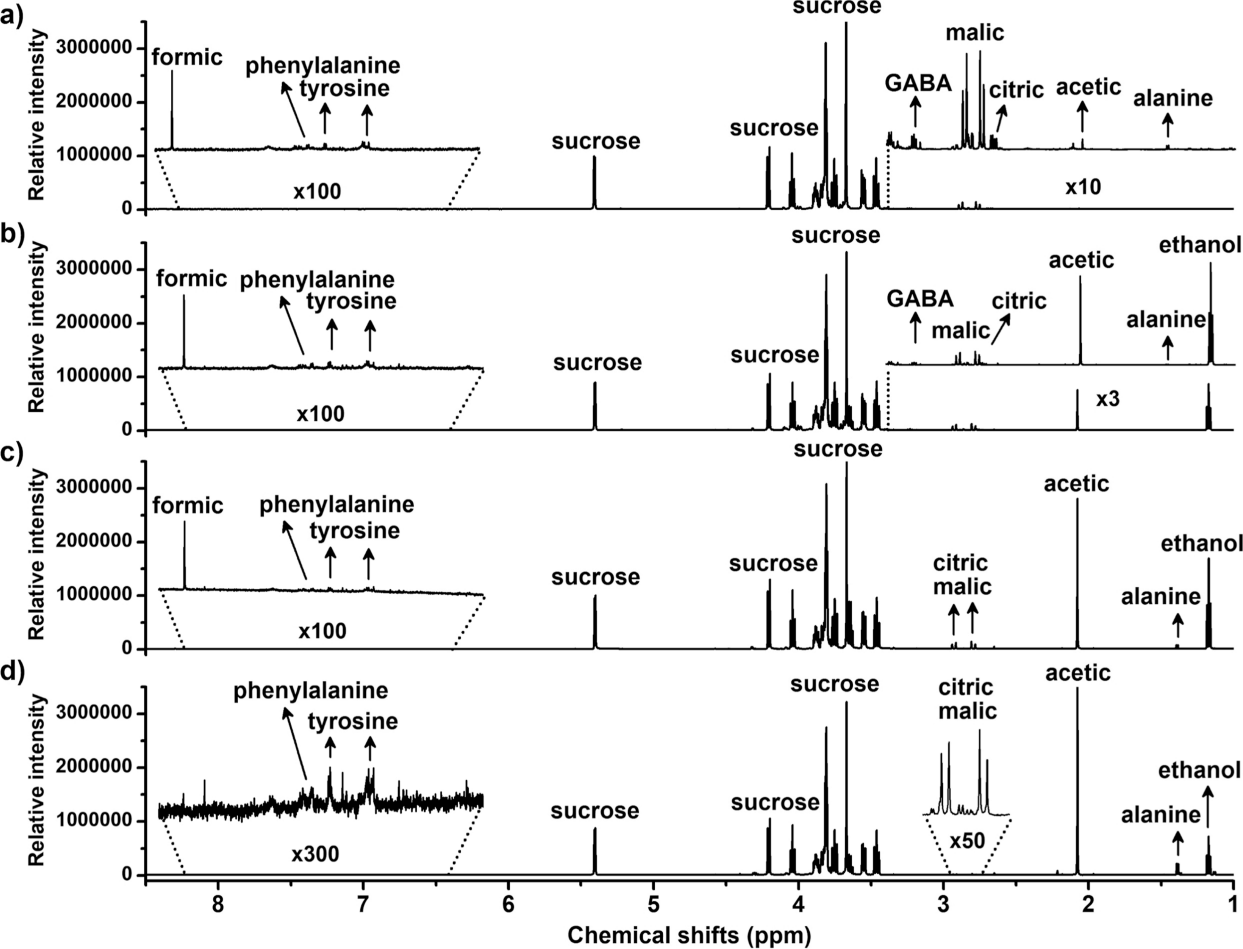
^1^H NMR spectra from pineapple juice-based kombucha during fermentation (0, 3, 5, and 7 days).

**Figure 4:**
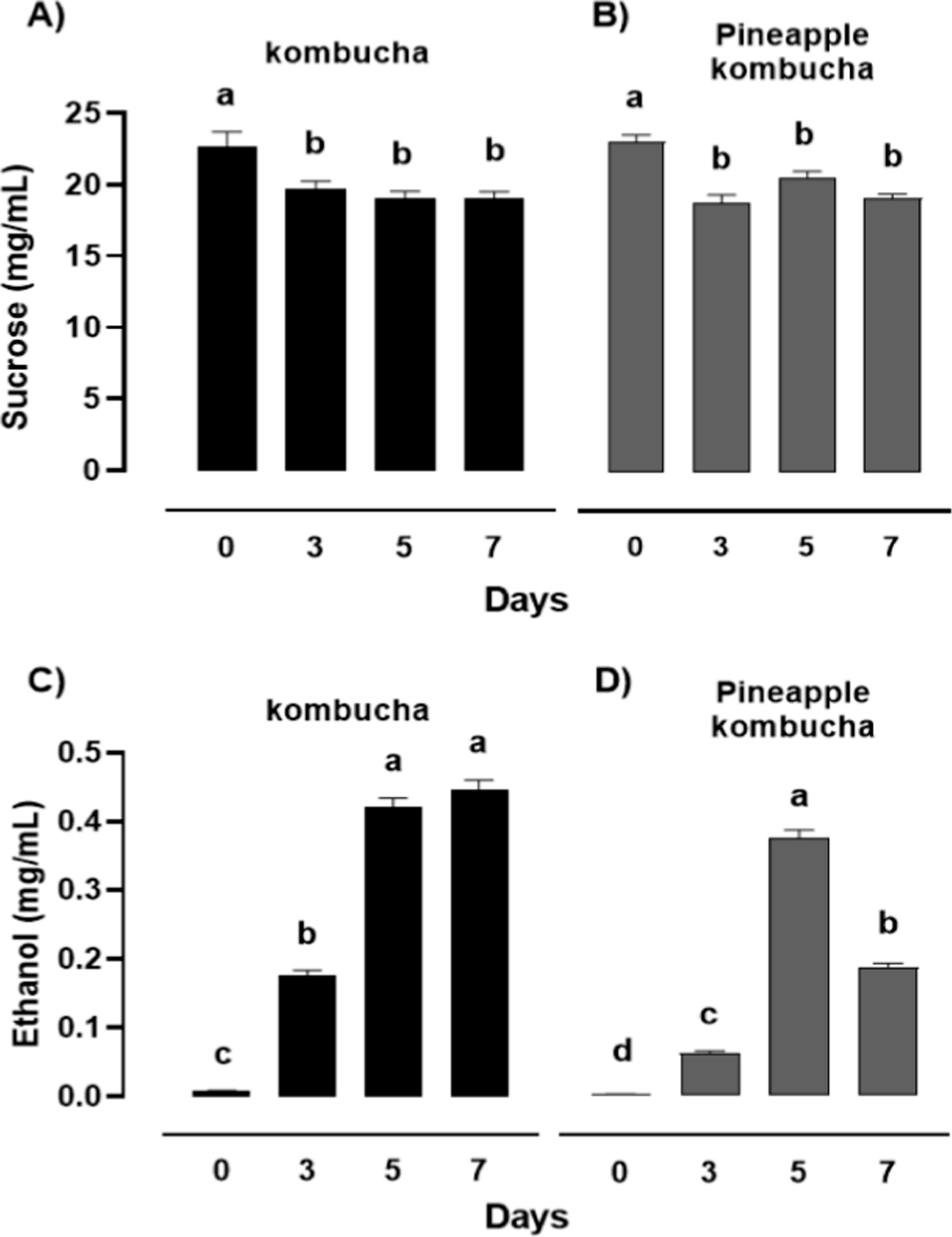
Quantitative variation of ethanol and sucrose determined by NMR in kombucha (black bars) (A,C) and pineapple juice-based kombucha (gray bars) (B,D) during fermentation (0, 3, 5, and 7 days). Bars represent mean values ±standard deviation (*n* = 3). Different lowercase letters above bars indicate statistically significant differences among fermentation times for each compound (*p* < 0.05, Tukey’s test).

In kombucha (Figure 4A), sucrose content showed a slight but consistent decrease from day 0 to day 7 (*p* < 0.05), indicating progressive utilization of the carbohydrate substrate by the yeast–bacteria consortium. Ethanol concentration (Figure 4C), initially low, increased sharply by day 5 (*p* < 0.05) and reached its maximum at day 7, confirming active alcoholic fermentation followed by the oxidation of ethanol to organic acids at later stages.^5^

In pineapple juice-based kombucha (Figure 4B), sucrose levels decreased less markedly (*p* < 0.05), as most sugars were already in the form of glucose and fructose in the fruit matrix. Nevertheless, ethanol accumulation followed a similar pattern to that observed in kombucha, with a pronounced rise by day 5 (*p* < 0.05) and stabilization thereafter (Figure 4D). These results illustrate the characteristic biphasic metabolism of kombucha fermentation, in which yeasts hydrolyze sucrose into glucose and fructose and convert them to ethanol and CO_2_ during the early anaerobic phase. Subsequently, acetic acid bacteria oxidize ethanol into acetic and gluconic acids, contributing to the acidification of the beverage.^2,3^

Importantly, ethanol formation is a transient indicator of microbial balance: moderate levels (≤0.5 g/L) enhance flavor complexity and contribute to the solvent extraction of polyphenols, while excessive accumulation may suppress bacterial activity and delay acid formation.^28^ The evolution of sucrose and ethanol contents corroborates the physicochemical data (reduction in TSS, decrease in pH, and increase in titratable acidity), indicating that the most active metabolic phase occurs between days 3 and 5.

### Changes in Amino Acids and Polyphenolic Compounds during Kombucha Fermentation

The quantitative variation of nitrogenous and phenolic compounds in green tea kombucha, determined by NMR spectroscopy across the fermentation period (0, 3, 5, and 7 days), is presented in Figure 5. It is noteworthy that theanine, caffeine, EGCG, and epicatechin gallate (ECG) were not detected in the pineapple juice-based kombucha at any fermentation time point, which is consistent with the fact that these compounds are naturally present in *C. sinensis* and are therefore exclusively associated with the green tea substrate.^31^ Theanine (Figure 5A) was detected throughout the fermentation period in green tea kombucha, of 0.008 ± 0.001 mg/mL at day 0, 0.005 ± 0.001 mg/mL at day 3, 0.009 ± 0.001 mg/mL at day 5, and 0.010 ± 0.002 mg/mL at day 7 (*n* = 3). Day 3 was statistically lower than days 0, 5, and 7 (Tukey’s test, *p* < 0.05), while days 0, 5, and 7 did not differ significantly from each other. Caffeine (Figure 5B) showed mean concentrations of 0.014 ± 0.004 mg/mL (day 0), 0.016 ± 0.002 mg/mL (day 3), 0.007 ± 0.003 mg/mL (day 5), and 0.009 ± 0.003 mg/mL (day 7). Days 0 and 3 were significantly higher than days 5 and 7, with a marked decline occurring between days 3 and 5 (Tukey’s test, *p* < 0.05).

**Figure 5:**
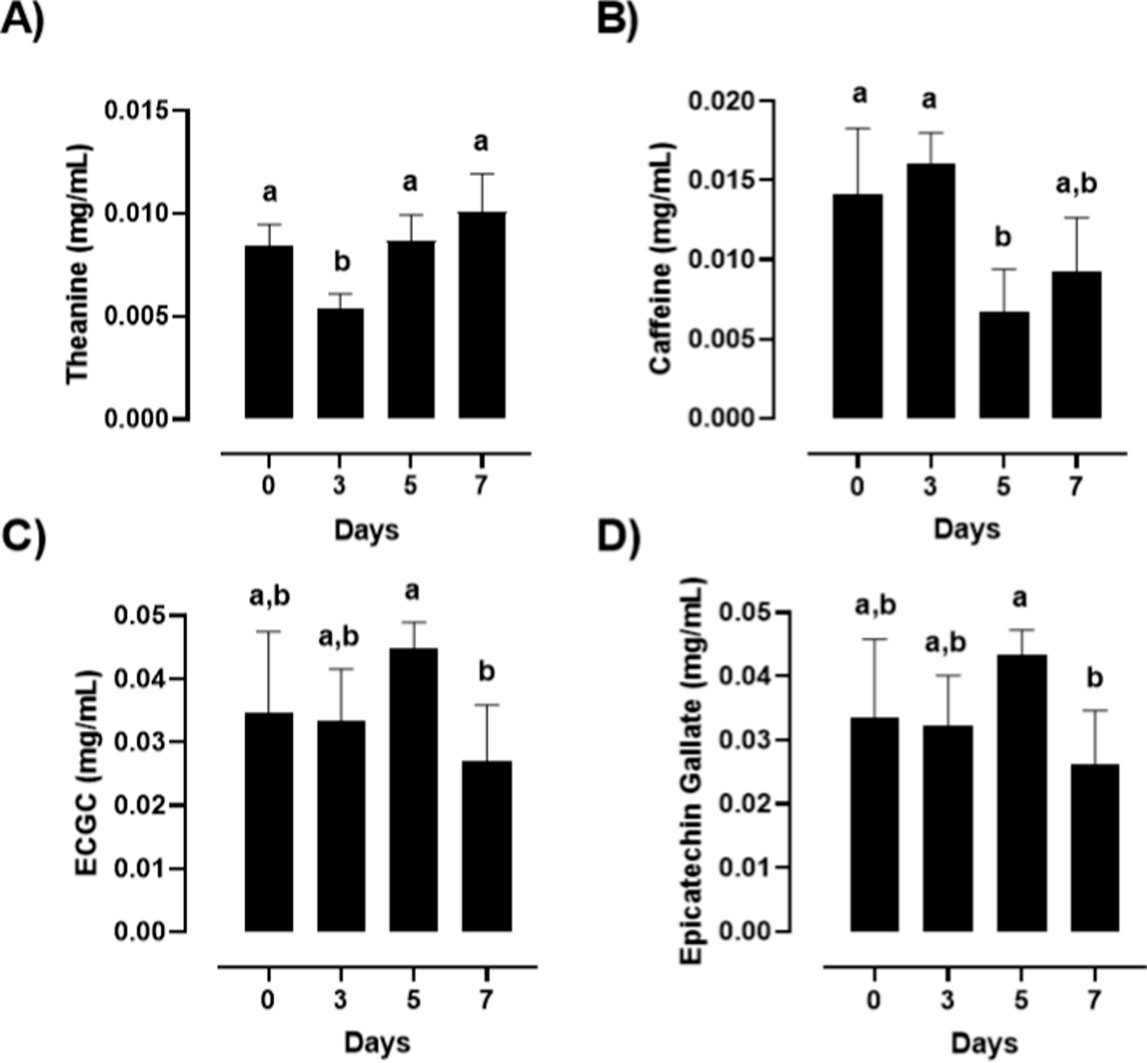
Quantitative variation of nitrogenous and phenolic compounds determined by NMR in green tea kombucha during fermentation (0, 3, 5, and 7 days). (A) Theanine; (B) caffeine; (C) epicatechin gallate (ECG); and (D) epigallocatechin-3-gallate (EGCG). Bars represent mean values ±standard deviation (*n* = 3). Different lowercase letters above bars indicate statistically significant differences among fermentation times within each beverage, while asterisks (*) denote significant differences between beverages for the same compound (*p* < 0.05, Tukey’s test).

Epigallocatechin-3-gallate (EGCG, Figure 5C) presented mean concentrations of 0.035 ± 0.013 mg/mL (day 0), 0.033 ± 0.008 mg/mL (day 3), 0.045 ± 0.004 mg/mL (day 5), and 0.027 ± 0.009 mg/mL (day 7). Day 5 was significantly higher than day 7, while days 0 and 3 did not differ significantly from either extreme (Tukey’s test, *p* < 0.05). Epicatechin gallate (ECG, Figure 5D) followed a similar pattern, with mean concentrations of 0.033 ± 0.012 mg/mL (day 0), 0.032 ± 0.008 mg/mL (day 3), 0.043 ± 0.004 mg/mL (day 5), and 0.026 ± 0.009 mg/mL (day 7). Day 5 was significantly higher than days 3 and 7, with days 0 and 3 again occupying an intermediate, nonsignificantly different position (Tukey’s test, *p* < 0.05).

The presence of theanine in green tea kombucha throughout the entire fermentation period is a relevant finding. l-Theanine was the most abundant amino acid in green tea (5.97 ± 0.36 μmol/g), and although it typically shows a marked decrease in fermented samples, a reduction in theanine in kombucha may result from its interaction with reducing substances produced during fermentation.^32^ The combination of l-theanine and other compounds produced from the fermentation of green tea extract by the kombucha starter generates a beverage with a unique taste and improved relaxation functionality.

From a health standpoint, theanine is a compound of considerable relevance: once in circulation, l-theanine can cross the blood–brain barrier and exert physiological effects in the central nervous system, promoting relaxation by influencing neurotransmitter activity, which helps lower blood pressure and reduce physiological responses to stress.^32^ Furthermore, l-theanine intake at standard dosages has been shown to increase alpha waves in healthy people and promote a state of psychophysical relaxation, with studies collectively suggesting improvement in cognitive reaction time and hints at increased cognitive function.^33^ The synergistic interaction between theanine and caffeine, both present in green tea kombucha, is also noteworthy, as l-theanine acts antagonistically against the stimulative effects of caffeine, producing a calming effect and, when taken together, the combination has been shown to improve cognitive performance and mood.^34^ The retention of theanine across the full seven-day fermentation period therefore reinforces the functional value of green tea kombucha as a relaxation-promoting beverage.

The fluctuation in caffeine levels throughout fermentation, with a peak at day 3 and a sharp decrease at day 5, is consistent with its dual role as both a nitrogen source for SCOBY microorganisms and a structurally stable alkaloid that resists full metabolic degradation. The fermentation process results in a beverage with less caffeine than regular tea because the SCOBY microbes utilize caffeine as a nitrogen source, which simultaneously leads to an increased production of the cellulosic mat.^35^ The partial recovery at day 7 likely reflects shifts in microbial community dynamics at later fermentation stages as previously reported in kinetic studies of kombucha fermentation. Beyond its well-known role as a central nervous system stimulant, caffeine in the context of a tea-based matrix is of particular interest due to its modulation by theanine, which attenuates jitteriness and promotes a state of alert calm rather than anxious excitation.^34^

The nonmonotonic behavior of EGCG and ECG, stable at days 0–3, peaking at day 5, and declining at day 7, reflects the complex enzymatic dynamics of the microbial consortium during fermentation. Microbial enzymes, including glucosidase, pectinase, xylanase, cellulase, and glucanase, can decompose polyphenol complexes into smaller polyphenol monomers, thereby transiently increasing measurable polyphenol content; conversely, the decline during extended fermentation may result from the activity of hydrolytic enzymes that break down polyphenols into metabolites used as nutrients for microbial metabolism.^2^ From a nutritional and health perspective, EGCG is particularly noteworthy. EGCG possesses anti-inflammatory, antioxidant, antifibrotic, and tissue-protective properties with therapeutic potential in cancer and neurological, cardiovascular, respiratory, and metabolic disorders; EGCG and ECG are the two most potent green tea catechins, with the galloyl moiety being responsible for their superior biological activity relative to other catechins.^36^ The maintenance of detectable EGCG and ECG levels throughout the entire fermentation period supports the antioxidant and anti-inflammatory potential of green tea kombucha as a functional beverage.

### Organic Acids Dynamics during Kombucha Fermentation

Figure 6 shows the quantitative variation of organic acids during kombucha fermentation (0, 3, 5, and 7 days), as determined by NMR. Acetic acid (Figure 6A) exhibited a marked and progressive increase throughout the fermentation process, rising from 0.003 ± 0.0005 mg/mL at day 0 to 0.168 ± 0.0072 mg/mL at day 3, 0.229 ± 0.0051 mg/mL at day 5, and reaching 0.303 ± 0.0076 mg/mL at day 7, with statistically significant differences among all time points (Tukey’s test, *p* < 0.05). In contrast, succinic acid (Figure 6B) decreased over time, from 0.005 ± 0.001 mg/mL at day 0 to 0.003 ± 2.72 × 10^–6^ mg/mL at day 3 and 0.001 ± 0.001 mg/mL at day 5, followed by a slight increase to 0.002 ± 0.001 mg/mL at day 7, suggesting its consumption during fermentation (Tukey’s test, *p* < 0.05). Gluconic acid (Figure 6C) showed an increasing trend up to day 5 followed by a decrease at day 7, indicating dynamic production and utilization during fermentation (Tukey’s test, *p* < 0.05). Gallic acid (Figure 6D) remained relatively stable, with values of 0.011 ± 0.003 mg/mL (day 0), 0.009 ± 0.002 mg/mL (day 3), 0.012 ± 0.001 mg/mL (day 5), and 0.012 ± 0.001 mg/mL (day 7), showing no significant variation. Additionally, formic acid (Figure 6E) presented a gradual increase from 0.0009 ± 0.001 mg/mL at day 0 to 0.0012 ± 0.001 mg/mL, 0.0014 ± 0.001 mg/mL, and 0.0020 ± 0.001 mg/mL at days 3, 5, and 7, respectively, although without statistically significant differences.

**Figure 6:**
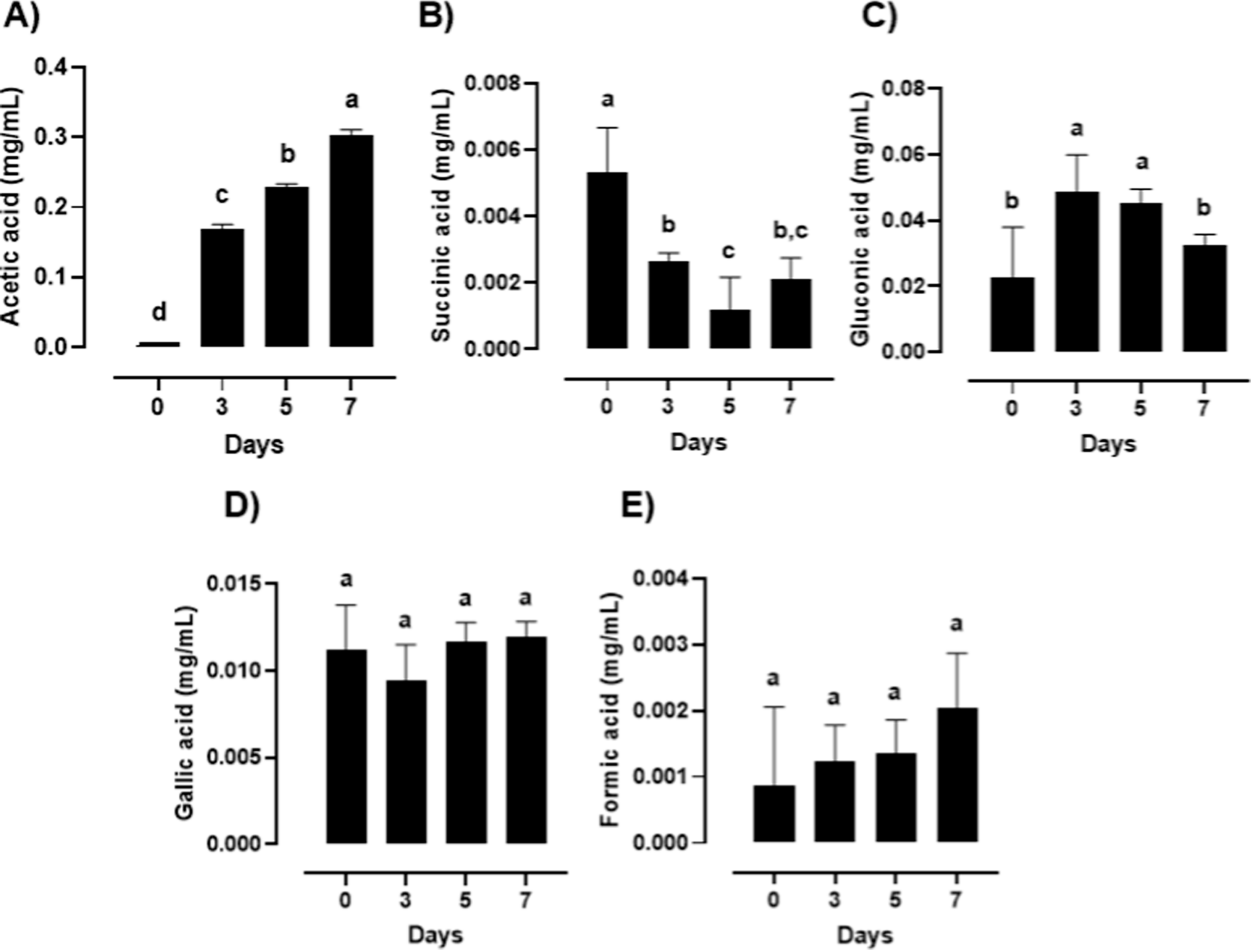
Quantitative variation of organic acids determined by NMR in kombucha during fermentation (0, 3, 5, and 7 days). Bars represent mean values ±standard deviation (*n* = 3). Different lowercase letters above bars indicate statistically significant differences among fermentation times for each compound (*p* < 0.05, Tukey’s test).

Overall, these results reflect the metabolic activity of the kombucha microbial consortium.^37^ The continuous accumulation of acetic acid is consistent with the oxidation of ethanol by acetic acid bacteria, contributing to the progressive acidification of the beverage.^37–39^ The reduction in succinic acid suggests its utilization as a metabolic intermediate, while the transient increase in gluconic acid may be associated with glucose oxidation followed by further microbial metabolism.^38,39^ The stability of gallic acid indicates a balance between its release from polyphenolic compounds and its potential transformation, whereas the slight increase in formic acid suggests its formation as a minor byproduct of microbial metabolism.^40–42^ Together, these findings highlight the dynamic biochemical changes occurring during kombucha fermentation.

### Metabolic Profile of Organic Acids and Amino Acids during Pineapple-Based Kombucha Fermentation

Figure 7 presents the quantitative variation of metabolites identified by NMR during the fermentation of pineapple-based kombucha (0, 3, 5, and 7 days). Acetic acid (Figure 7A) showed a pronounced and statistically significant increase during fermentation, rising from 0.004 ± 0.0008 mg/mL at day 0 to 0.168 ± 0.0073 mg/mL at day 3, 1.315 ± 0.0112 mg/mL at day 5, and reaching 1.947 ± 0.0360 mg/mL at day 7. Succinic acid (Figure 7B) exhibited a gradual increase, with values of 0.003 ± 0.000 mg/mL at day 0, 0.003 ± 0.000 mg/mL at day 3, 0.004 ± 0.000 mg/mL at day 5, and 0.009 ± 0.001 mg/mL at day 7 (Tukey’s test, *p* < 0.05). Malic acid (Figure 7C) increased from 0.060 ± 0.018 mg/mL at day 0 to a maximum of 0.096 ± 0.004 mg/mL at day 5, followed by a slight decrease to 0.075 ± 0.004 mg/mL at day 7.

**Figure 7:**
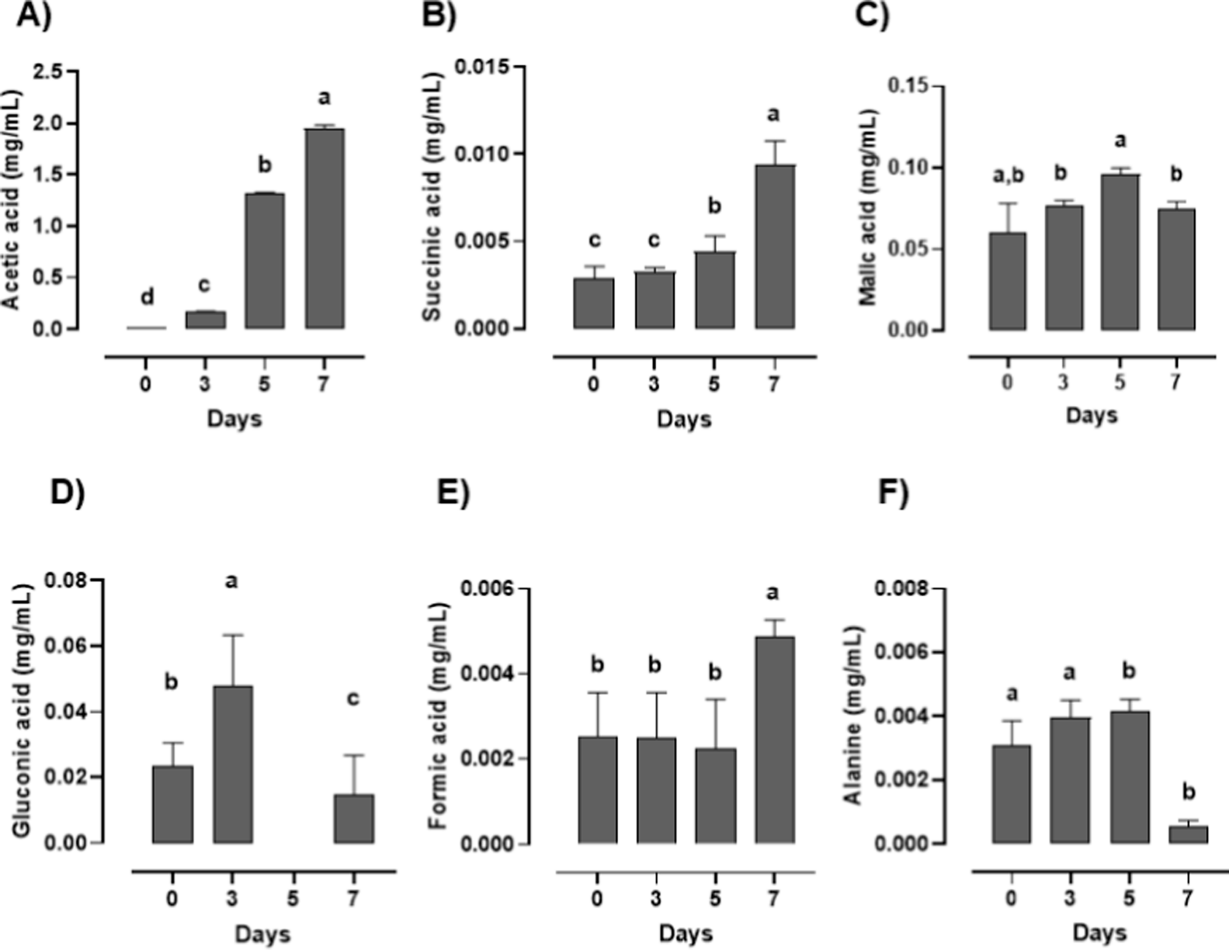
Quantitative variation of organic acids identified by NMR in pineapple-based kombucha during fermentation (0, 3, 5, and 7 days). Bars represent mean values ±standard deviation (*n* = 3). Different lowercase letters above bars indicate statistically significant differences among fermentation times for each compound (*p* < 0.05, Tukey’s test).

Gluconic acid (Figure 7D) initially increased from 0.023 ± 0.007 mg/mL at day 0 to 0.048 ± 0.016 mg/mL at day 3, then decreased sharply at day 5 (0.000 ± 0.000 mg/mL), with a partial recovery at day 7 (0.015 ± 0.012 mg/mL) (Tukey’s test, *p* < 0.05). Formic acid (Figure 7E) remained relatively stable during the early stages, with values of 0.0025 ± 0.0010 mg/mL (day 0), 0.0025 ± 0.0011 mg/mL (day 3), and 0.0023 ± 0.0012 mg/mL (day 5), followed by a significantly (Tukey’s test, *p* < 0.05) increase at day 7 (0.0049 ± 0.0004 mg/mL). Alanine (Figure 7F) showed slight variation, with values of 0.003 ± 0.0008 mg/mL at day 0, 0.004 ± 0.0005 mg/mL at day 3, 0.004 ± 0.0004 mg/mL at day 5, and a significant decrease (Tukey’s test, *p* < 0.05) to 0.001 ± 0.0002 mg/mL at day 7.

Overall, the results demonstrate intense metabolic activity during pineapple kombucha fermentation, particularly highlighted by the substantial accumulation of acetic acid, indicating active oxidation of ethanol by acetic acid bacteria.^37^ The increase in succinic and malic acids suggests their involvement in microbial metabolic pathways, while the dynamic profile of gluconic acid reflects its production from glucose followed by rapid utilization.^37^ The late increase in formic acid may be associated with secondary metabolic processes or stress-related pathways. The reduction in sucrose confirms its consumption as a primary carbon source, whereas the decrease in alanine at later stages suggests its utilization as a nitrogen source.^37–39^ Together, these findings reinforce the complex biochemical transformations occurring during fermentation and the influence of the pineapple matrix on metabolite dynamics.

### Antioxidant Activity by ABTS Assay

The antioxidant activity determined by the ABTS assay showed significant differences between the beverages, while no significant changes were observed over the fermentation time within each group (Figure 8).

**Figure 8:**
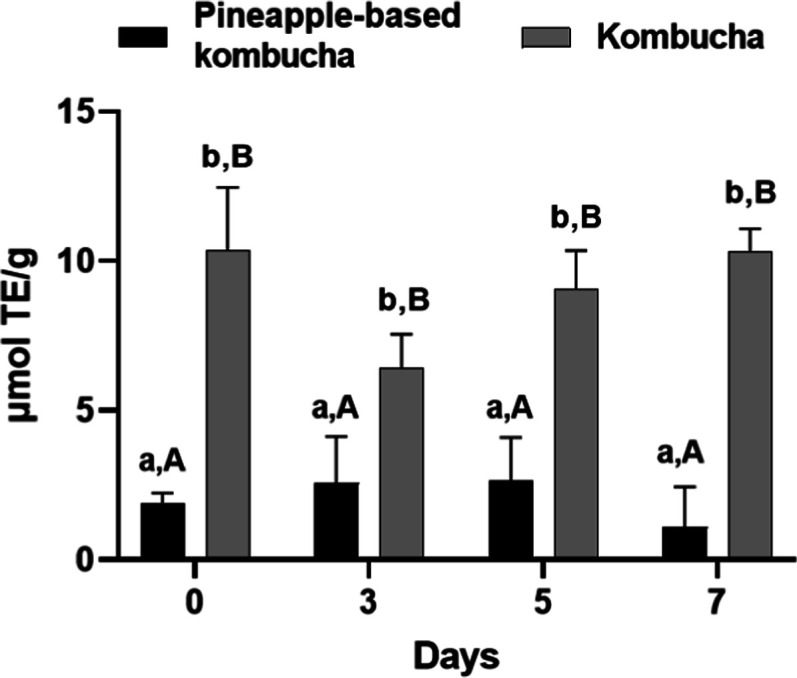
Antioxidant activity determined by the ABTS assay in pineapple-based kombucha and green tea kombucha during fermentation (0, 3, 5, and 7 days). Results are expressed as mean ± standard deviation (*n* = 3). Different lowercase letters indicate significant differences among fermentation times within the same beverage, while different uppercase letters indicate significant differences between beverages at the same fermentation time (*p* < 0.05, two-way ANOVA followed by Tukey’s multiple comparisons test).

For pineapple-based kombucha, the mean antioxidant activity was 1.903 μmol TE/g on day 0, increasing to 2.567 μmol TE/g by day 3, 2.943 μmol TE/g on day 5, and reaching 3.000 μmol TE/g by day 7. In contrast, traditional kombucha exhibited significantly higher antioxidant activity, with a mean of 10.363 μmol TE/g on day 0, decreasing to 6.403 μmol TE/g on day 3, then rising to 9.067 μmol TE/g on day 5, and finally reaching 10.327 μmol TE/g by day 7.

Comparatively, green tea kombucha showed significantly higher antioxidant capacity than pineapple-based kombucha at all fermentation times, as indicated by different uppercase letters. These results suggest that while both types of kombucha exhibit varying antioxidant activity throughout fermentation, traditional kombucha consistently demonstrates higher levels compared with pineapple-based kombucha.

The higher antioxidant activity observed in traditional kombucha compared with pineapple-based kombucha can be primarily attributed to the rich presence of polyphenols and flavonoids in tea.^2^ Compounds like epigallocatechin gallate (EGCG) and other catechins, which are abundant in traditional kombucha, are known for their strong antioxidant properties.^2,3^ In contrast, while pineapple contains some antioxidants, its overall concentration and variety of beneficial compounds are generally lower than those found in tea.

Additionally, the fermentation process of traditional kombucha produces various organic acids and metabolites that further enhance its antioxidant capacity. The stability of these compounds tends to be greater in traditional kombucha, while the antioxidants in pineapple may degrade more quickly during fermentation.^2,3,8^ This difference, along with the synergistic effects of multiple active compounds in traditional kombucha, contributes to its superior antioxidant activity compared with that of pineapple-based kombucha.

These findings align with Jakubczyk et al.^31^ who noted that tea type significantly affects antioxidant potential due to varying polyphenol compositions, with green tea being a rich source of flavonoids, contributing to higher antioxidant activity. Hsieh et al.^40^ further demonstrated that both tea type and fermentation time influence antioxidant capacity, showing an initial increase, followed by stabilization. Our results support this, as no significant variations were noted between days 0 and 7, suggesting a stable antioxidant phase.

In contrast, Syahviqra and Sanuddin^41^ reported that pineapple kombucha exhibited higher antioxidant activity than traditional kombucha; however, our study did not confirm this. This discrepancy may stem from differences in active compound compositions, as traditional kombucha benefits from tea-derived polyphenols with stronger radical scavenging abilities. Additionally, differences in analytical methods (DPPH vs ABTS) and fermentation conditions could influence antioxidant measurements. Notably, Mfopa et al.^42^ reported significant increases in ABTS radical scavenging activity over extended fermentation periods, with peak activity observed at 21 days, highlighting the crucial role of the fermentation time.

Taken together, these findings reinforce that the antioxidant capacity of kombucha is influenced by multiple factors, including substrate composition, fermentation time, and the analytical method. While fruit-based substrates such as pineapple can enhance antioxidant properties under certain conditions, tea, especially green tea, remains a more consistent and potent source of antioxidant compounds in the kombucha systems.

## Conclusion

The fermentation of green tea- and pineapple-based kombucha displayed distinct yet complementary biochemical and functional characteristics. NMR and physicochemical analyses demonstrated a consistent increase in acetic, gluconic, and succinic acids, alongside a significant pH reduction and dynamic transformations in amino acids and polyphenols. Green tea kombucha preserved higher concentrations of key bioactive compounds such as catechins (EGCG and ECG) and theanine, which underpin its superior antioxidant capacity as corroborated by ABTS assay results. In contrast, pineapple kombucha exhibited accelerated acidification and higher total acid content, driven by its abundant simple sugars and inherent organic acids. Despite some antioxidant activity gains during fermentation, pineapple kombucha’s overall radical scavenging ability remained lower due to its limited polyphenol content and faster degradation of phenolic compounds. The integration of these findings identifies 5 days of fermentation as the optimal point to achieve a favorable balance among acidity, pH, and bioactive compound retention. Controlled fermentation terminating at this stage ensures microbiological safety, desirable sensory characteristics, and enhanced functional potential, enabling the tailored development of novel kombucha beverages with diversified health benefits derived from both tea and fruit substrates.

## Supplementary Material



## Notes

NMR parameters from the identification of the compounds in different kombucha samples; ^1^H-^1^H COSY and ^1^H-^13^C HSQC from a representative juice-based kombucha sample (PDF)
