# Supplementary material for: Optimization of Kombucha Fermentation from Green Tea and Pineapple Juice: Chemical, Physicochemical, and Bioactive Profiles for Functional Beverage Development
Source: ACS Omega. 2026 May 14;11(20):30197–207. doi: 10.1021/acsomega.6c03893 (PMC13216971; doi:10.1021/acsomega.6c03893)
Supplement: Supplementary file 1 [file ao6c03893_si_001.pdf]

# Optimization of kombucha fermentation from green tea and pineapple juice: chemical, physicochemical, and bioactive profiles for functional beverage development

Socorro Vanesca Frota Gaban[1*], Samylla Sáthilla Queiroz Nogueira[1], Francisco Marlon Mota Marques[1], Chiara Porro[3], Elenilson G. Alves Filho[1], Lorena Mara A. Silva[2], Kirley M. Canuto[2].

[1]Department of Food Engineering, Federal University of Ceara, Bloco 858, Campus do Pici, CEP 60440-900, Fortaleza, Ceará, Brazil.

[2]Embrapa Agroindústria Tropical, Rua Doutora Sara Mesquita, n. 2270, Pici, CEP 60511-110 Fortaleza-CE, Brazil.

[3]Department of Clinical and Experimental Medicine, University of Foggia, 71121 Foggia, Italy.

*E-mail of the corresponding author: vanescafm@hotmail.com

Table S1 describes the characterization parameters from the NMR analyses of the organic composition of the control kombucha and pineapple juice-based kombucha during fermentation (0, 3, 5, and 7 days). Figures S2 and S3 presents de $^{1}H$-$^{1}H$ COSY and $^{1}H$-$^{13}C$ HSQC from a representative juice-based kombucha sample.

**Table S1.** The NMR parameters from the identification of the compounds in different kombucha samples: chemical structure; experimental and reference $^{1}H$ and $^{13}C$ chemical shifts [21-24].

| *Structures* | *δ $^{1}H$ (multip.* J in Hz)* | *δ $^{13}C$ (HSQC)* | *δ $^{1}H$ ref.* | *δ $^{13}C$ ref.* |
|---|---|---|---|---|
| | *AMINO ACIDS* | | | |
| *Alanine* 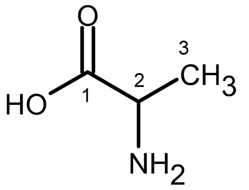 | 2 - (o)<br>3 - 1.49 (*d* 7.2) | 52.4<br>19.9 | 3.90 (*q* 7.3)<br>1.52 (*d* 7.3) | 53.4<br>19.1 |
| *GABA* 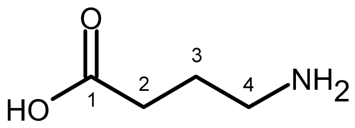 | 4 – 3.06 (*m*)<br>3 – 1.97 (*m*)<br>2 – 2.50 (*m*) | 42.0<br>25.3<br>34.0 | 2.99 (*t* 7.6)<br>1.88 (*qui* 7.6)<br>2.28 (*t* 7.6) | 42.2<br>26.3<br>37.1 |
| *Theanine* 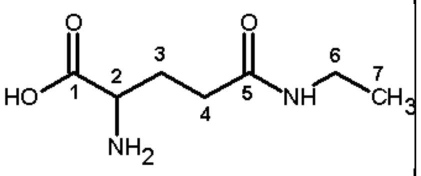 | 2 –<br>3 –<br>4 –<br>6 –<br>7 – | | 3.79 (t)<br>2.11 (*m*)<br>2.21 (t)<br>1.22 (t)<br>3.32 (q) | 56.4<br>29.7<br>33.8<br>34.6<br>12.6 |
| *Tyrosine* 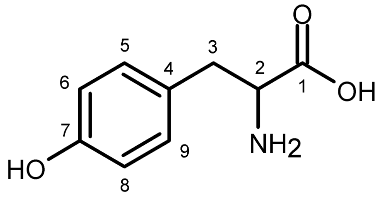 | 6.8 – 6.92 (*m*)<br>5.9 – 7.53 (*m*) | 119.3<br>133.6 | 6.89 (*m*)<br>7.19 (*m*) | 118.9<br>133.5 |

| | | | | |
|---|---|---|---|---|
| *Phenylalanine*<br>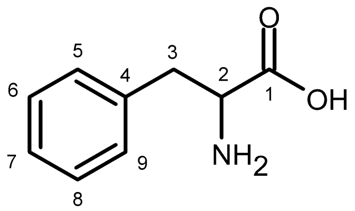 | 5.9 – 7.33 (*m*)<br>6.8 – 7.43 (*m*)<br>7 – 7.38 (*m*) | 132.5<br>132.4<br>130.8 | 7.32 (*d* 6.98)<br>7.42 (*m*)<br>7.37 (*m*) | 132.1<br>131.8<br>130.4 |

*ORGANIC ACIDS*

| | | | | |
|---|---|---|---|---|
| *Acetic*<br>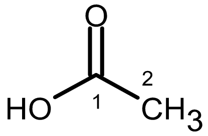 | 2 - 1.94 (*s*) | 27.0 | 1.90 (*s*) | 26.1 |
| *Citric*<br>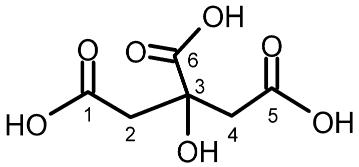 | 2 - 2.62 (o)<br>4 - 2.74 (o) | 47.7<br>47.7 | 2.52 (*d* 15.8)<br>3.66 (*d* 15.8) | 48.6<br>48.6 |
| *Formic*<br>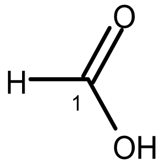 | 1 - 8.46 (*s*) | no | 8.46 (*s*) | 173.9 |
| *Gallic acid*<br>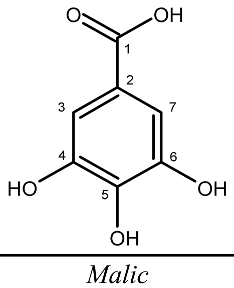 | 3 – 7.17 | 113.4 | 7.04 | 112.2 |
| *Malic*<br>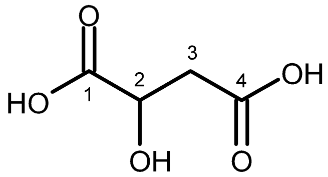 | 3 - 2.40 (*m*)<br>3 - 2.70 (*m*)<br>2 - 4.31 (*m*) | 45.2<br>45.2<br>73.2 | 2.68 (*dd*)<br>2.85 (*dd*)<br>4.28 (*m*) | 45.5<br>45.5<br>73.2 |

*CARBOHYDRATES*

| | | | | |
|---|---|---|---|---|
| *Sucrose*<br>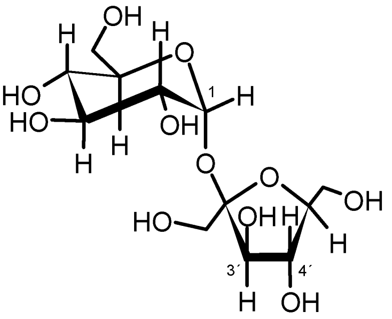 | 1 - 5.41 (*d* 3.70)<br>2 - 3.56 (o)<br>3 - 3.76 (o)<br>4 - 3.48 (o)<br>5 - 3.85 (o)<br>6 - 3.82 (o)<br>1' - 3.82 (o)<br>2' - 3.89 (o)<br>3' - 4.05 (*m*)<br>4' - 4.22 (*m*)<br>6' - 3.68 (*m*) | 95.1<br>74.1<br>75.5<br>72.3<br>75.5<br>63.1<br>65.2<br>84.3<br>77.0<br>79.3<br>64.5 | 5.44 (*d* 3.80)<br>3.89-3.57 (*m*)<br>n<br>n<br>n<br>n<br>n<br>n<br>4.08 (*t* 8.40)<br>4.24 (*d* 9.0)<br>n | 94.7<br>73.5<br>75.0<br>71.8<br>74.9<br>62.8<br>64.0<br>83.7<br>76.6<br>79.0<br>65.0 |

*OTHER COMPOUNDS*

| | | | | |
|---|---|---|---|---|
| *Caffeine*<br>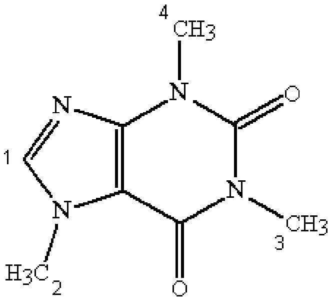 | 1 - 7.89 (*s*)<br>2 - 3.97 (*s*)<br>3 - 3.55 (*s*)<br>4 - 3.43 (*s*) | 144.3<br>35.0<br>30.6<br>30.0 | 7.82 (s)<br>4.00 (*s*)<br>3.59 (*s*)<br>3.41 (*s*) | 141.4<br>33.7<br>29.3<br>28.0 |
| *Ethanol*<br>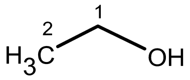 | 1 – 3.62<br>2 – 1.14 | 60.6<br>20.8 | 3.64<br>1.17 | 60.3<br>19.6 |
| *Epigallocatechin (EGC)*<br>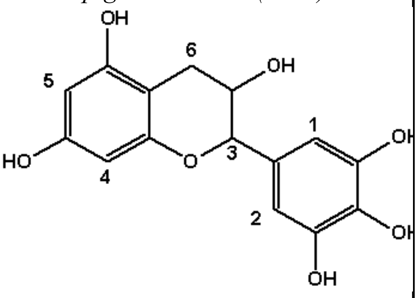 | 1,2 – 6.61 (*s*)<br>3 – 4.90 (o)<br>4 – 6.05 (*d* 2.30)<br>5 – 6.09 (*d* 2.30)<br>6 – 2.85 (*m*) | 105.4<br>no<br>99.8<br>100.2<br>no | 6.50 (*s*)<br>5.00<br>5.99 (*d* 2.28)<br>5.76 (*d* 2.28)<br>2.83 | 107.2<br>80.1<br>95.6<br>96.4<br>28.6 |
| *Epigallocatechin-3-gallate (EGCG)*<br>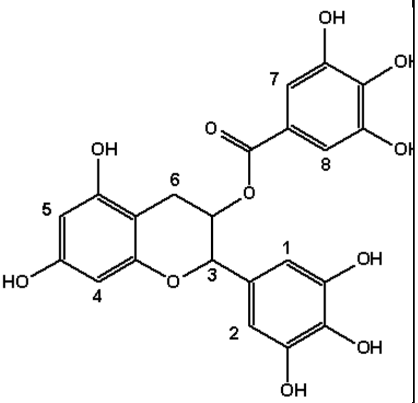 | 1,2 – 6.57 (*s*)<br>3 – 4.90 (o)<br>4 – 6.08 (*d* 2.30)<br>5 – 6.12 (*d* 2.30)<br>6 – 2.94 (*m*)<br>7,8 – 6.78 (*s*) | 105.4<br>no<br>99.8<br>100.2<br>no<br>112.7 | 6.50 (*s*)<br>5.00 (n)<br>5.99 (*d* 2.28)<br>5.76 (*d* 2.28)<br>2.83 (n)<br>6.76 (*s*) | 107.2<br>80.1<br>95.6<br>96.4<br>28.6<br>110.0 |

*s* – simplet; *d* – duplet; *t* – triplet; *q* – quadruplet; *quin* – quintet; *dd* – double of duplets; *dt* – double of triplets; o – overlapping signal; n – no information; no – not observed.

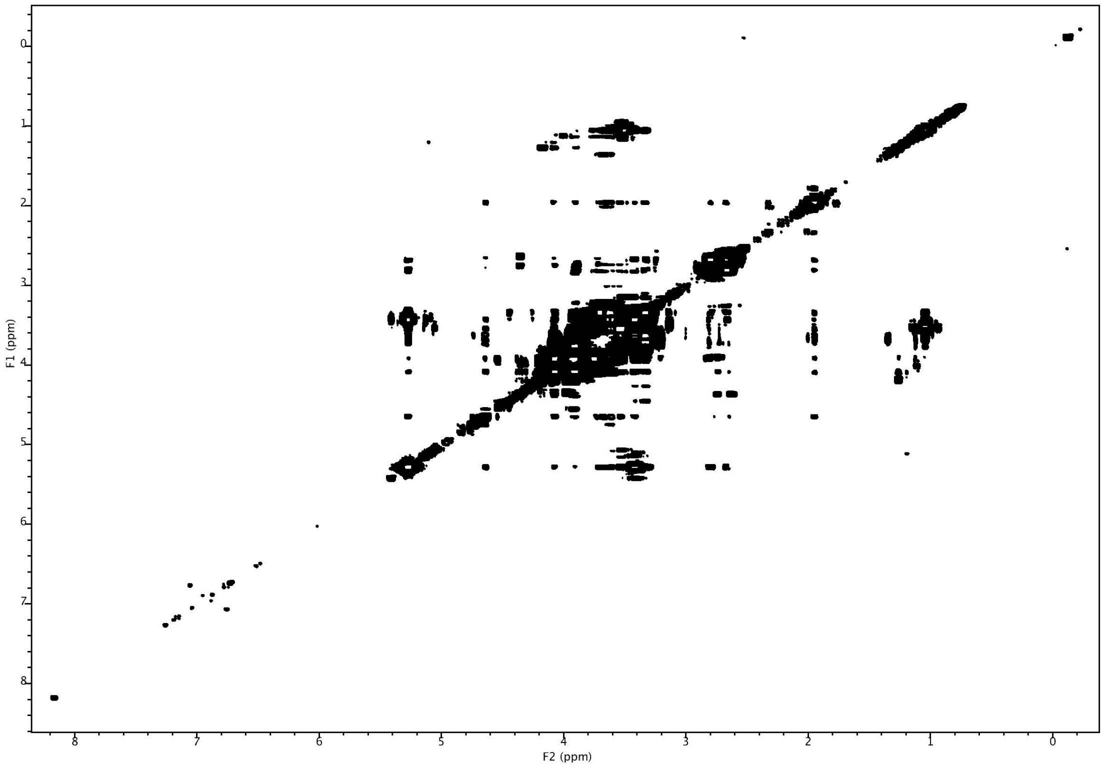


**Figure S2.** $^{1}$H-$^{1}$H COSY and $^{1}$H-$^{13}$C HSQC from a representative juice-based kombucha sample (after 5 fermentation days).

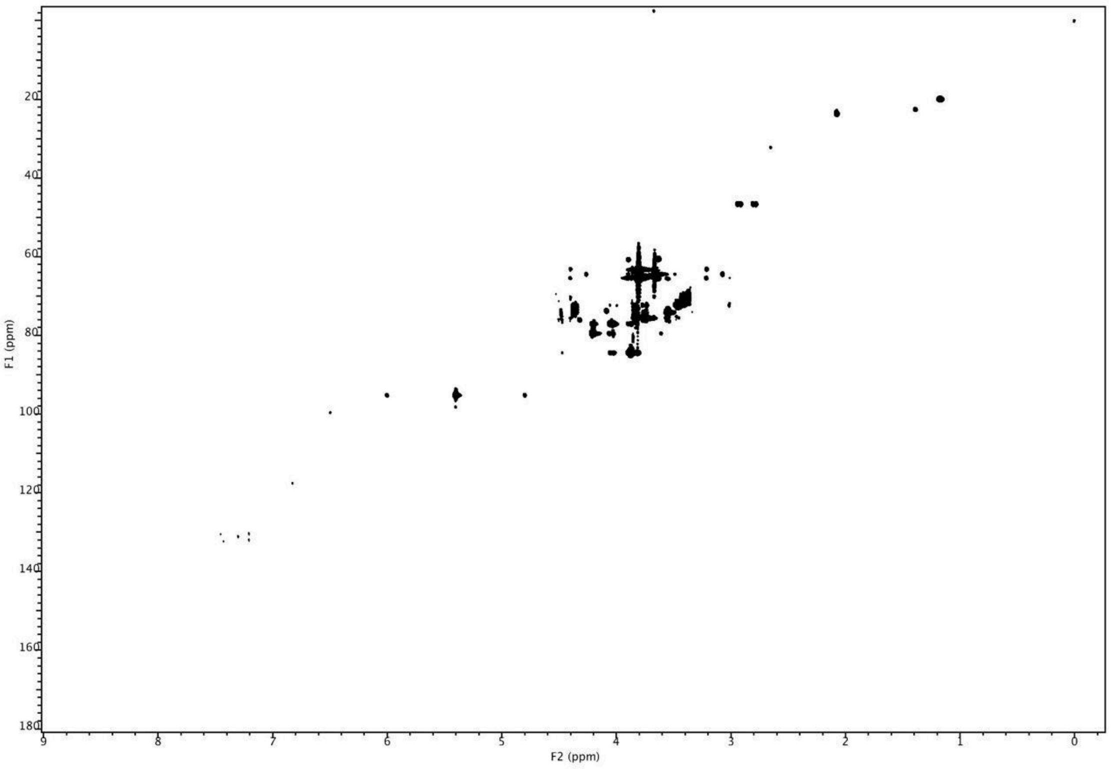


**Figure S3.** $^{1}H$-$^{13}C$ HSQC from a representative juice-based kombucha sample (after 5 fermentation days).
